# High-Performance Biocomputing in Synthetic Biology–Integrated Transcriptional and Metabolic Circuits

**DOI:** 10.3389/fbioe.2019.00040

**Published:** 2019-03-11

**Authors:** Angel Goñi-Moreno, Pablo I. Nikel

**Affiliations:** ^1^School of Computing, Newcastle University, Newcastle upon Tyne, United Kingdom; ^2^The Novo Nordisk Foundation Center for Biosustainability, Technical University of Denmark, Kongens Lyngby, Denmark

**Keywords:** biocomputing, synthetic biology, metabolic engineering, boolean logic, genetic circuits, metabolic networks

## Abstract

Biocomputing uses molecular biology parts as the hardware to implement computational devices. By following pre-defined rules, often hard-coded into biological systems, these devices are able to process inputs and return outputs—thus *computing* information. Key to the success of any biocomputing endeavor is the availability of a wealth of molecular tools and biological motifs from which functional devices can be assembled. Synthetic biology is a fabulous *playground* for such purpose, offering numerous genetic parts that allow for the rational engineering of genetic circuits that mimic the behavior of electronic functions, such as *logic gates*. A grand challenge, as far as biocomputing is concerned, is to expand the molecular hardware available beyond the realm of genetic parts by tapping into the host metabolism. This objective requires the formalization of the interplay of genetic constructs with the rest of the cellular machinery. Furthermore, the field of metabolic engineering has had little intersection with biocomputing thus far, which has led to a lack of definition of metabolic dynamics as computing basics. In this perspective article, we advocate the conceptualization of metabolism and its motifs as the way forward to achieve *whole-cell biocomputations*. The design of merged transcriptional and metabolic circuits will not only increase the amount and type of information being processed by a synthetic construct, but will also provide fundamental control mechanisms for increased reliability.

## Biocomputing

Computation can be broadly defined as the formal procedure by which input information is processed according to pre-defined rules and turned into output data. Since this definition does not specify the type of *information* and *rules* involved in the process, it is applicable to electronic devices as well as to biological systems. In other words, biological systems *do* perform computations. While the computational ability of biological matter has been explicitly described a number of times along the twentieth century (Bennett, 1982), it was Leonard Adleman who showed the feasibility of implementing human-defined computations with molecular (i.e., genetic) hardware (Adleman, 1994). Although the discussion on what would be the equivalent of computer hardware and software in biological systems is still largely open (Danchin, 2009), the term *hardware* in this article identifies any physical, tangible component (e.g., nucleic acids or metabolites) in a cell. On this first example of biocomputation, Adleman physically encoded an instance of the Hamiltonian path problem (a well-known mathematical problem in graph theory) in DNA strands, and solved it *in vitro* by using routine molecular biology methods. A bacterial computer (i.e., an *in vivo* computer), would solve an instance of the same problem 15 years later (Baumgardner et al., 2009). By the end of last century, Weiss et al. (2002) showed that synthetic regulatory networks could be conceptualized *in vivo* as a series of Boolean logic gates–the key device of cellular computers. This novel conceptual framework set the start of a frantic wave of electronic-inspired bioengineering in synthetic biology. Additionally, these seminal works also shifted the inspiration within the biocomputing community drastically, from mathematics and computer science to electronic engineering.

## Whole-Cell Biocomputations

Cells are able to process input information in many different and intricate ways. For the sake of clarity, in this article we propose to group the processing of information into two types of computing (i.e., *genetic* and *metabolic*) depending on the nature of the input and components thereof. To date, most of the biocomputing developments in synthetic biology dealt almost exclusively with genetic material and parts. This type of approach limits the scope of the potential synthetic biocomputations that can be executed, since a number of important resources are not being utilized. In a challenging paper entitled “It's the metabolism, stupid!,” de Lorenzo (2015) suggested that “the interplay of DNA and metabolism is […] akin to that of politics and economy. Both realms drive their own autonomous agendas and obviously influence each other.” In a similar fashion, the field of *heterotic computing* (Kendon et al., 2015) advocates the use of various types of computing that merge the strengths of individual types into more powerful, *heterotic* devices.

### Synthetic Biology as an Active Biocomputing Field

**Figure 1:**
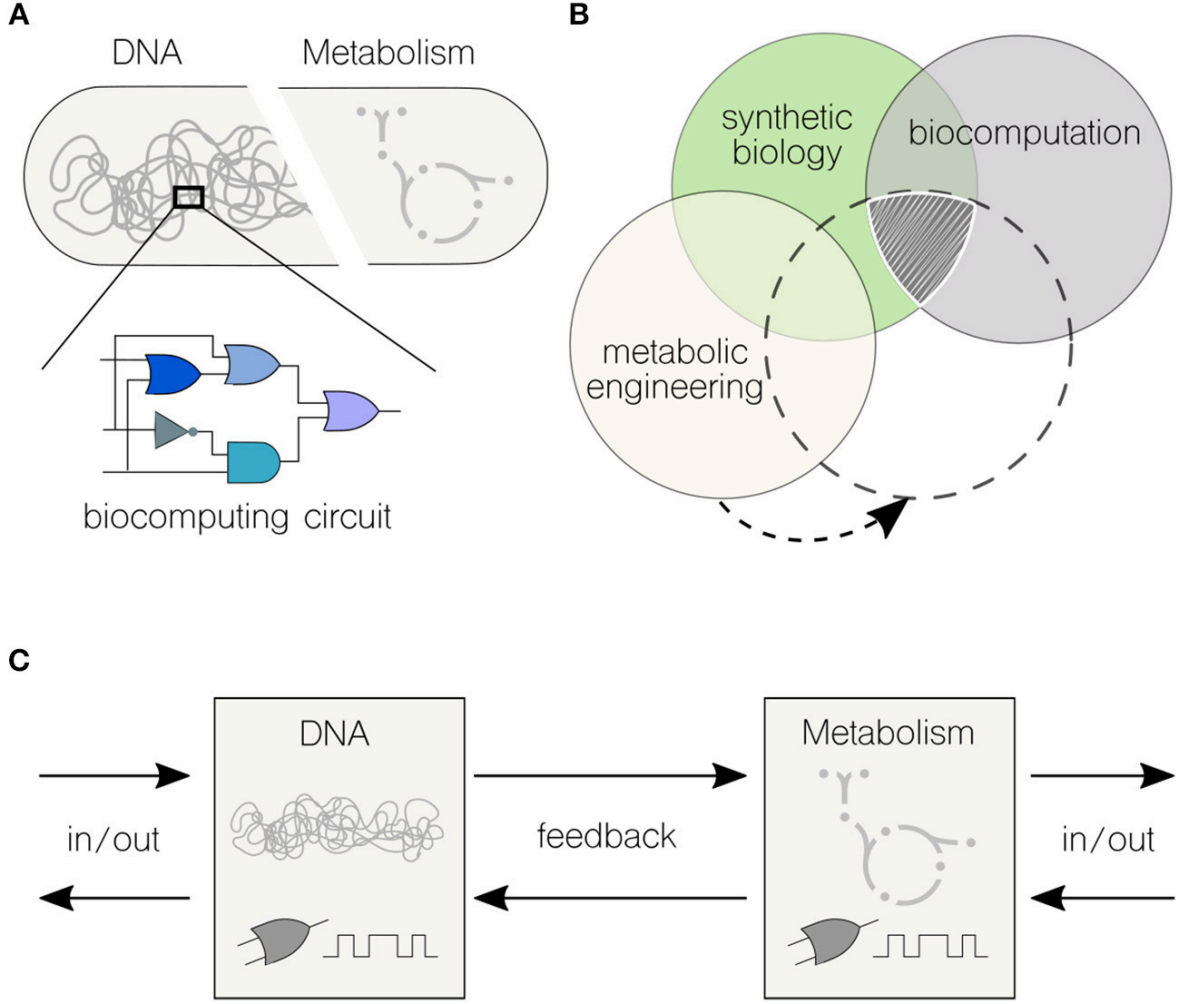
Interfacing genetic and metabolic processes for high-performance biocomputations. **(A)** Biocomputing circuits are typically encoded into genetic material. Synthetic biology provides an extensive toolkit of genetic parts and devices that are assembled to build combinatorial (and even sequential) logic circuits. The metabolic environment where the circuit *runs* is often overlooked when it comes to formalize logic motifs. **(B)** The expanding field of biocomputation intersects synthetic biology. Genetic logic circuits have been central to synthetic biology since the formal inception of the discipline. Thus, far, there is no obvious exploitation of this type of biocomputation for metabolic engineering–there is, however, enough synergy between the three disciplines to find an overlapping (sub)field. **(C)** Information processing flows in merged transcriptional and metabolic circuits. Both transcriptional and metabolic networks are able to sense external inputs and yield output responses; the feedback from one layer to the other can effectively communicate information.

Boolean logic is central to the field of computing. Therefore, the design and implementation of Boolean logic functions in cells—typically encoded into genetic material (Figure 1A)—is key to the development of synthetic biology approaches rooted on biocomputing (Amos and Goñi-Moreno, 2018). The engineering of a genetic *toggle* switch (Gardner et al., 2000) and an *oscillator* (Elowitz and Leibler, 2000) in *Escherichia coli* at the onset of the twenty-first century had set the start of what is a very active field nowadays. Over the last (almost) 20 years, a number of circuits have been successfully engineered in living cells, such as logic gates mentioned above (Wang et al., 2011), counters (Friedland et al., 2009), multiplexers (Moon et al., 2011), adders (Ausländer et al., 2012), and memories (Bonnet et al., 2013). Inspired by computer science, distributed computations have also been designed and build in multicellular systems by modifying cell-cell communication programmes (Goñi-Moreno et al., 2011, 2019; Regot et al., 2011). From solving relatively simple mathematical problems to compute intricate Boolean logic operations, biological systems have proved to be a powerful platform for tackling applications that are restricted to traditional “silicon-based” computer technologies, such as diagnosis, bioproduction, and bioremediation.

Synthetic biocomputing circuits are growingly more complex and accurate every day, mostly due to endless efforts in improving the genetic toolkit (Silva-Rocha et al., 2013; Martínez-García et al., 2014; Durante-Rodríguez et al., 2018), mathematical methods (Church et al., 2014; Goñi-Moreno and Amos, 2015) and design procedures (Goñi-Moreno and Amos, 2012; McLaughlin et al., 2018) for the so-called *design-build-test-learn* synthetic biology cycle (Goñi-Moreno et al., 2016). There are, nevertheless, major challenges on the genetic computing front (Goñi-Moreno, 2014; Manzoni et al., 2016), such as the urgent need for standardization of components, measurements, and information (Myers et al., 2017; Fabre and Sonnenschein, 2019). As long as synthetic biology claims to be a true engineering discipline, such a standardization problem must be tackled without delay to enable *bona fide* modularity and predictability of genetic circuits (Vilanova et al., 2015). Altogether, the implementation of biocomputations using genetic material is driven by an excellent scientific momentum at the present time.

### Metabolic Engineering as a *Potential* Biocomputing Field

While there is a phenomenal potential for development, the metabolic aspect of computation has not been explored to the same degree as it has been implemented *via* genetic circuits (Figure 1B). This fact arises from a still-limited knowledge on the complexity of metabolic networks even in the so-called “model” organisms (Benedetti et al., 2016; Calero and Nikel, 2019). Nielsen and Keasling (2016) have recently stressed the presence of metabolic networks with hard wired, tightly regulated lines of communication in virtually all living cells–which are inherently difficult to manipulate but, as the very definition implies, offer an unique opportunity for engineering multi-level computations. In the same way synthetic biology uses genetic parts and devices to build complex systems with pre-defined behaviors, metabolic networks are characterized by some (more or less conserved) principles that can be used for re-purposing biochemical nodes. The bowtie model of central metabolism indicates that the core biochemistry of the cell includes the biochemical transformations necessary for the synthesis of the 12 known essential biomass precursors (Noor et al., 2010). This architecture requires a high level of regulation, especially at the level of gene transcription (Kochanowski et al., 2017). Is precisely at this intersection between cellular processes that biocomputing could play a role in re-programming the metabolic machinery of cells.

### High-Performance Biocomputing

Natural cellular pathways are rarely based on genetic or metabolic activities alone. Thus, the concept of heterotic computing (i.e., the coordination between different types of computing), is intrinsic to biological systems. However, synthetic circuits are not often exploiting the full computational power of the cellular machinery. Although the type of processes is very different, the cooperation between them could pave the way to a new generation of whole-cell circuits with enhanced abilities. This aspect is what we refer to as *high-performance biocomputing*.

Against this background, Figure 1C shows the flows of information in high-performance biocomputations. A first challenge would be to describe what in computing are called *primitives*, which are the simplest elements with which software programs are built upon. This will result in a set of well-characterized genetic and metabolic units (e.g., coding sequences and metabolic reactions) and motifs (e.g., oscillations and switches), including types of inputs and outputs for each computing end. Although current efforts are individually tackling this challenge in either the genetic (Nielsen et al., 2016) or metabolic (Sánchez-Pascuala et al., 2017) fronts, there is still the issue that genetic and metabolic units must be *plugged* together to allow information flow in both directions. This connectivity will enable the direct modification of genetic motifs by the action of their metabolic counterparts (and vice-versa). Depending on the specific process, and the type of information being computed, either of the two ends could return the desired output.

The increasing focus on the interplay between genetic and metabolic networks (Shlomi et al., 2007; Kumar et al., 2018) is resulting in a revolution of metabolic engineering driven by the core principles of synthetic biology. Not only molecular tools are actively being developed (Keasling, 2012; Nikel and de Lorenzo, 2018), but also control strategies to engineer genetic circuits are being increasingly exploited for the regulation of metabolism in a pre-defined fashion (Oyarzún and Stan, 2013; Chen and Liu, 2018; Moser et al., 2018). The foundations of high-performance biocomputing are therefore established and ready to benefit from the input of the computing community. Yet, a solid representation framework is needed to fully realize this purpose.

## A Unified Representation Framework

Boolean logic is a way to abstract the underlying mechanistic details of a device into its high-level functional performance. By doing so, gene expression can be abstracted into *ON*/*OFF* states (i.e., either the gene is, or is not, being expressed under a given environmental condition) regardless of the particularities the gene of interest might have. Even in the case of radical analog fluctuations in gene expression, the *ON*/*OFF* abstraction still provides an useful conceptual framework (García-Betancur et al., 2017; Goñi-Moreno et al., 2017). However, when it comes to implementation, the Boolean abstraction needs to be complemented by a dynamical analysis of the components at stake. For example, the time-scales with which genetic and metabolic interactions occur can potentially be very different. Therefore, the dynamic analysis of individual reactions is as fundamental as the functional representation of the system as a whole.

**Figure 2:**
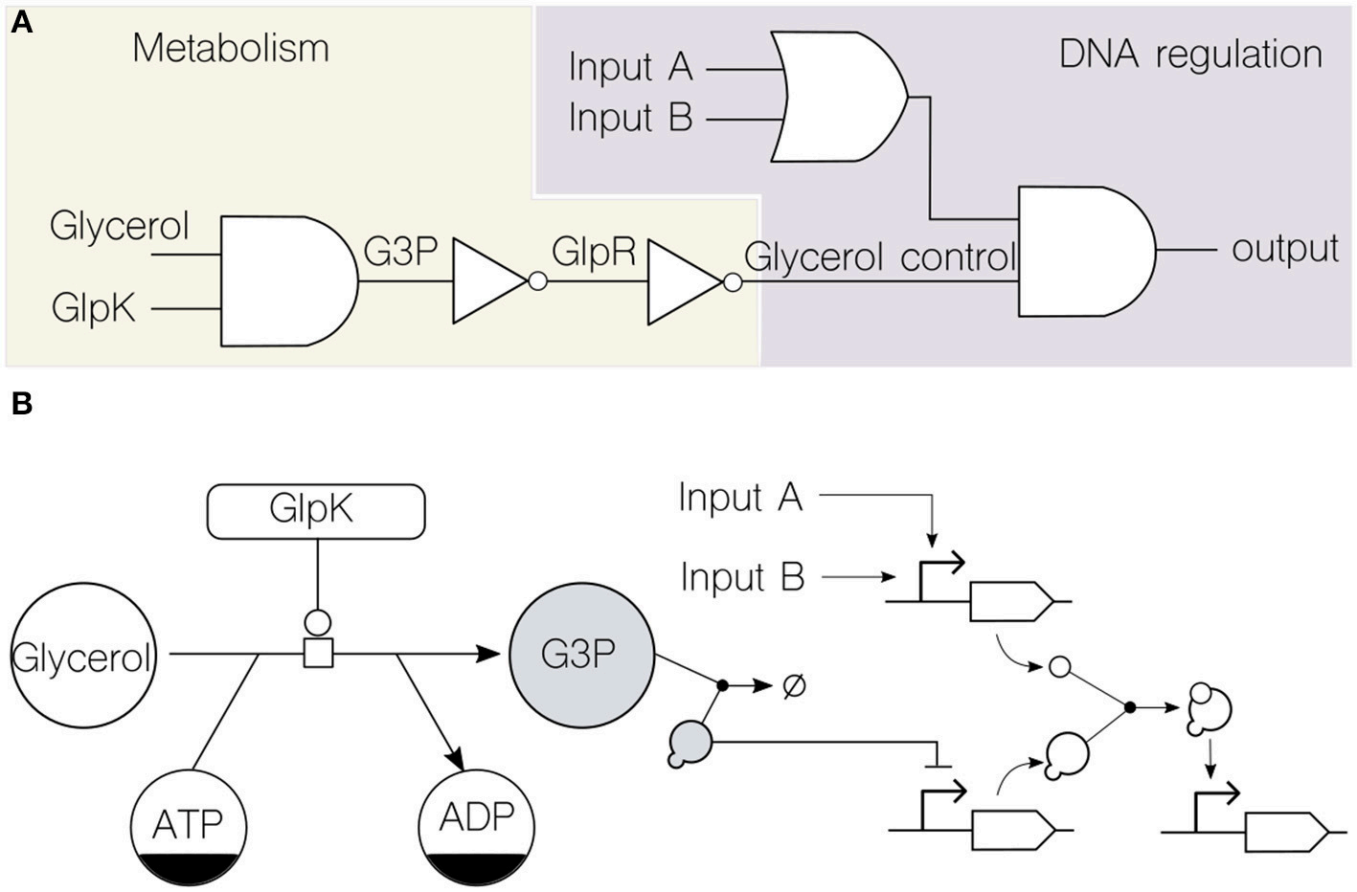
Formal representation of merged transcriptional and metabolic circuits. **(A)** Circuit formalization into Boolean functions (i.e., logic gates) assists the combination of metabolism and DNA regulation. Using the known transcriptional and metabolic network that rules glycerol consumption in the soil bacterium *P*. *putida* KT2440 (Nikel et al., 2014) as an example, the circuit depicts the role of glycerol as an input along with other signals (*inputs A* and *B*) typically used in transcriptional logic. Note that the flow of information is bidirectional, since the metabolic GlpK input (a key enzyme involved in glycerol consumption) can be modified by the genetic circuit. This top-level logic design enables the abstraction of details about the type of substrate used by providing a unified computing framework. **(B)** The same glycerol circuit is formalized through the adoption of existing representation standards: the *Systems Biology Graphical Notation* (SBGN) for metabolic networks and the *Synthetic Biology Open Language* (SBOL) visual for genetic circuits. The two shaded components, the key metabolite glycerol-3-phosphate (G3P) and the transcriptional repressor GlpR, constitute the physical link that merge both computing layers.

To illustrate this point, we discuss a case of merged genetic and metabolic circuitry integration in a platform bacterium. Figure 2A shows a logic-gate representation of a simple merged transcriptional and metabolic circuit in the soil bacterium *Pseudomonas putida* KT2440. This device merges state-of-the-art DNA regulatory circuitry (Nielsen et al., 2016) with dynamics that are far beyond DNA reach: the metabolic ability of the cells to catabolize glycerol (Nikel et al., 2015). In this way, the circuit output depends not only in the upstream computation of typical genetic inputs (generic *inputs A* and *B* in the diagram) but also in the metabolic dynamics of glycerol uptake. The link that enables the functioning of the circuit is the transcriptional repressor GlpR, which somewhat encodes information about the metabolic state of the cell (the action of GlpR on the cognate *glp* gene cluster is relieved by the metabolite glycerol-3-phosphate, G3P) and acts on a specific promoter. Note that virtually any other signaling molecule or transcription factor that feeds the final genetic AND logic gate can be inserted downstream of this promoter. Moreover, any regulatory step in the circuit can be connected back to, e.g., the key enzyme GlpK (essential for glycerol processing) thus providing feedback control from the genetic to the metabolic side of the device. As a result, the combinatorial genetic logic circuit is now linked to the physiological state of the cell concerning the dynamics of carbon source uptake, which can be both read and controlled.

Using a lower, more specific, layer of representation, the *Systems Biology Graphical Notation* (SBGN) (Le Novère et al., 2009) and the *Synthetic Biology Open Language* (SBOL) (Galdzicki et al., 2014) were used to formalize the circuit (Figure 2B) for both metabolic and genetic parts, respectively. This helps identifying the link, which in this case is formed by the interaction between a metabolite (G3P) and a transcriptional repressor (GlpR)—thereby merging the metabolic and genetic layers of regulation in the bacterial cell (Figure 2B).

We recently coined the term *metabolic widget* to refer to such merged circuits (Chavarría et al., 2016). The metabolic machinery of the cell, often referred to as the *context* when focusing on genetic logic, offers powerful resources that can greatly improve current biocomputations. Far from trying to avoid the context, the framework proposed herein is taking full advantage of it, which can lead to widgets that assist more complex and accurate pre-defined processes of information. The adoption of such a configuration will have a double impact by providing essential information about both the metabolic and genetic wiring of the cell while taking full advantage of these interactions for re-programming core cellular functions.

From a broader perspective, evolution has shaped intricate cellular processes that merge both genetic and metabolic networks; yet, human-defined biocomputations rarely make use of both computing types. We advocate for taking this path into account in order to access and exploit the high-performance biocomputing power intrinsic to natural systems, empowering the design-build-test-learn cycle to entirely new directions.

## Author Contributions

Both authors have made a substantial, direct and intellectual contribution to the work, and approved it for publication.

### Conflict of Interest Statement

The authors declare that the research was conducted in the absence of any commercial or financial relationships that could be construed as a potential conflict of interest.
